# Paper-based microextraction tool for the *in vitro* and eco-friendly detection and degradation of malathion pesticide from soybean seeds

**DOI:** 10.1039/d5ra06061c

**Published:** 2025-12-02

**Authors:** Archana Kumari, Harshika Poojary, Chiranjit Ghosh, S. Balaji

**Affiliations:** a Department of Biotechnology, Manipal Institute of Technology (MIT), Manipal Academy of Higher Education, Manipal (MAHE) Karnataka 576104 India chiranjit.ghosh@manipal.edu s.balaji@manipal.edu

## Abstract

To increase agricultural productivity, malathion is widely used as a low-cost organophosphate pesticide for soybean cultivation to protect crops from insects. The unregulated use of malathion facilitates its entry into the food chain, resulting in harmful effects on human health. Conventional sample preparation techniques are limited due to their multi-step processes and excessive solvent consumption. Here, paper-based microextraction strips were developed with phytase enzyme and divinylbenzene (DVB) and polydimethylsiloxane (PDMS) polymers for the simultaneous preconcentration of malathion and the degradation of the pesticides into less toxic phosphorodithioic acid (PDA) byproducts. The sampling strips were tested with a soybean seed matrix after spiking with 500–10000 ng mL^−1^ of malathion and finally analyzed by triple quad gas chromatography mass spectrometry. The method achieved a limit of detection of ∼82 ng mL^−1^ for malathion quantification, ensuring the practical applicability of the strips as per regulatory limits (2000 ng mL^−1^ by the Codex Alimentarius and European Union regulations). The dual-functionality-based paper strips provide a single analytical platform for capturing the malathion pesticide and degrading this pesticide into non-toxic byproducts. The method exhibited good linearity and a high greenness score, confirming the eco-friendliness and efficiency of the sample preparation technique for the determination of pesticides. Therefore, this simple, disposable enzyme-coated paper strip could be useful for the detection and remediation of malathion from soybean seeds in a green way.

## Abstract 2

Thin-film microextraction tool for the detection and degradation of malathion pesticide from soybean seeds.

## Graphical abstract

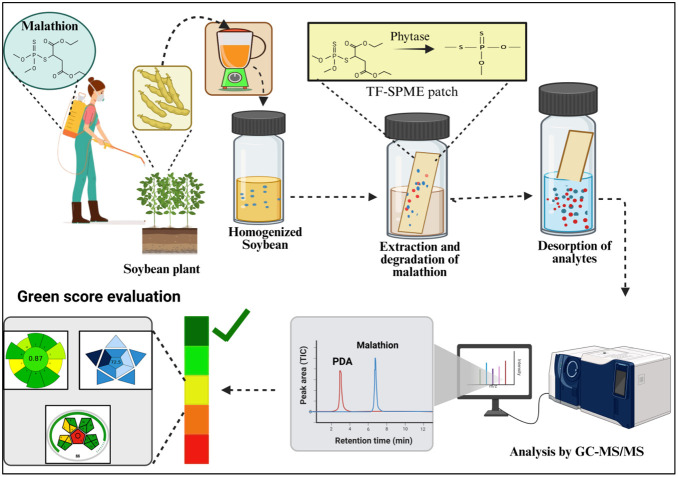

## 1 Introduction

Soybean (*Glycine max* L.) is a nutritious and highly valuable legume due to its ample protein content and other health-promoting attributes. It is one of the most significant plant-based protein sources in human nutrition.^1^ Soybeans are also processed to make other foodstuffs, including soy milk, tofu, soy flour, and soy sauce, and these are consumed worldwide. In food technology, soy products are important for their functional properties, including fat replacement, gelling, and emulsification.^2,3^ The global production of soybeans has increased significantly over the last decades due to their nutritional value and agricultural versatility.^4^ However, the patterns of soybean consumption around the world are different. A high intake of soy products has been reported in Asian countries. It is very common among vegetarians who consume soy as their dominant source of protein.^2^

However, the increased demand for food has led to the use of agrochemicals in the cultivation of crops. The soybean production worldwide, especially in developing countries, strongly relies on the use of pesticides to enhance agricultural yield. The unregulated use of those pesticides is associated with severe environmental and human health hazards.^5,6^ Agrochemicals, particularly pesticides, aid in boosting crop production by guarding against pests during pre- and post-harvest seasons.^7^ The most common family of pesticides is organophosphates (OPs), which include malathion, methyl parathion, diazinon, endosulfan, dimethoate, chlorpyrifos, quinalphos, profenofos, and monocrotophos.^8^ Nevertheless, such compounds always tend to leave residual contaminants in the food, and this can pose a serious health hazard. The health consequences of pesticide exposure include both short-term effects, such as vomiting, skin discomfort, and headaches, and long-term effects, such as kidney damage, reproductive health concerns, cancer, and even death.^9–11^

Among OPs, malathion stands out as one of the most extensively used pesticides due to its relatively lower acute toxicity compared to other OPs.^12^ Its wide utilization has contributed to environmental dispersion and excessive exposure through ingestion, inhalation, or direct contact with the skin.^13^ Malathion residues have been detected in human biological samples, such as blood, urine, and hair, including those of pregnant women, raising a serious concern about the potential toxicity of this pesticide.^14,15^ Although the use of malathion was approved in agriculture, the excess use of this pesticide poses great concern. As per the report of Codex Alimentarius and European Union regulations, the Maximum Residue Limit (MRL) for malathion is 2 mg kg^−1^ (2000 ng mL^−1^). Therefore, there is a pressing need to develop a potential technique for the fast and efficient detection of malathion and the simultaneous remediation of the extracted pesticide for environmental safety.

Pesticide residues from the edible portion of fruits and vegetables should be actively monitored to reduce the harmful levels of toxic substances in agricultural products.^16^ Several analytical sample preparation methods have been developed for the detection of pesticides, including liquid–liquid extraction (LLE) and solid-phase extraction (SPE).^17,18^

To overcome the limitations associated with traditional techniques, solid-phase microextraction (SPME) was introduced by Pawliszyn in the 1990s.^19^ It is an alternative approach to reduce the amount of extractants to microliters and decrease the analysis time.^20–22^ To improve the extraction efficiency, thin-film solid-phase microextraction (TF-SPME) was derived as an advanced form of SPME by Pawliszyn and co-workers in 2003.^23^ The working principle of both the SPME and TF-SPME techniques involves the adsorption and desorption of analytes on an adsorbent-coated phase of solids.^24,25^ These methods are either solvent-free or require a minimal amount of solvents, and therefore, these sample preparations are considered eco-friendly and sustainable.^26,27^ For instance, Limam *et al.* applied SPME fibres for the determination of phenol, methyl phenols, chlorophenols and bisphenol in water samples.^28^ Nevertheless, the lack of strength and the small surface area of traditional SPME fibres have limited their widespread applicability in analytical fields, especially in trace-level environmental analysis. Piri-Moghadam *et al.* used GC-MS and SPME to detect organophosphates in water samples. To overcome the fragility issue, TF-SPME with enhanced surface area, flexible, and thermally stable polymer-covered films is an exceptional choice for a wide range of applications.^29,30^ In comparison with exhaustive extraction techniques, it mainly extracts free analytes without interfering with the biological systems.^31,32^ However, the high cost of commercial patches has limited their applications for regular food quality checks.

In recent years, Green Analytical Chemistry (GAC) concepts have gained significant popularity as a step towards minimizing the consequences of analytical methods on the environment.^33^ GAC is directly associated with the reduction of hazardous chemicals, waste minimization and efficiency of the analysis step.^34^ To evaluate the eco-friendliness of analytical techniques, several analytical greenness metric methodologies, including Analytical Greenness Metric Approach and Software (AGREE),^35,36^ Blue Applicability Grade Index (BAGI),^37^ and Green Analytical Procedure Index (GAPI),^38^ have been developed. These analytical greenness metrics allow the evaluation of the sustainability of the analytical procedures quantitatively and objectively.^39^

In this research, we developed a paper-based microextraction patch for the detection and degradation of malathion from soybean seed samples. The paper patches were coated with divinylbenzene (DVB) and the enzyme phytase as a malathion-degrading agent. While DVB provided strong hydrophobic interactions for efficient malathion extraction, phytase enabled the *in situ* enzymatic hydrolysis of the pesticide directly on the patch surface. This integrated approach allowed us the simultaneous extraction and transformation of malathion into its less toxic byproduct. Unlike conventional techniques, such as SPE and LLE, the proposed method minimized solvent usage, shortened the extraction time, and offered a potential approach for the fast detection of malathion pesticide from soybean seeds. Furthermore, the use of a paper-based substrate made this tool cost-effective and environmentally sustainable compared to the commercial TF-SPME devices. This study demonstrated the feasibility of an enzyme-assisted, cost-effective, and disposable paper strip for the estimation of pesticides from a complex matrix, including soybean seeds. It also introduces a potential approach for the degradation of malathion on the same analytical platform.

## 2 Materials and methods

### 2.1 Materials

The commercial malathion was purchased from the Katyayini MAL50 brand. The phytase from wheat enzyme was bought from Sigma Aldrich (Bangalore, India). We took normal commercially available A4-size sheet papers (Copy Gold A4 sheet, GSM-75, size 21 × 29.7 cm). Extra pure grade (98%) acetonitrile was purchased from LOBA Chemicals (Mumbai, India), whereas hexane was purchased from Sigma Aldrich (Bangalore, India). PDMS, air-tight vials and 40 mL glass vials with septum and caps were purchased at Sigma-Aldrich (Bangalore, India). Sigma-Aldrich supplied monomer DVB. LOBA Chemicals supplied the initiator AIBN. The soybeans were acquired from the local markets. Amcor (mode PM-996) was used as the source of Parafilm. All the glassware used in this study were procured from Borosil.

### 2.2 Instruments

To quantify the pesticide, we utilized a gas chromatograph-mass spectrometer (GC/MS-QP2020 NXSHIMADZU, Shimadzu Corp., Tokyo, Japan). In this case, a column SH-I-5Sil MS, 0.25 mm inner diameter and 30 m length, was used. Helium was used as a carrier gas with a column flow rate of 1.2 mL min^−1^. The GC oven was set in an automatic temperature program consisting of a temperature of 50 °C for 2 min, a 6 °C ramp up to 280 °C, then a hold of 1 min, an increase of 15 °C up to 320 °C and finally a hold of 2 min. The ion source temperature (EI) was held constant at 230 °C. A uniform coating of 70 µm was applied at room temperature using an automated film applicator (Elcometer 4340, 44 Manchester, UK). The purchased magnetic stirrer (model: 2MLH) and vortex shaker CM-101 Plus were purchased by REMI. The REMI CIS-18 Plus shaking incubators were used for the extraction of compounds from the soybean matrix. The centrifugation procedure was done using the Eppendorf 5804R centrifuge.

### 2.3 Statistical analysis

Parametric and nonparametric data were analyzed using OriginPro 2024b (Origin Lab Corporation, USA) software. Here, we reported the data in the form of the mean ± SE. To determine the reproducibility of the experimental data at different concentration ranges, we presented all the mean data after three-point repetitions. We used the calibration curve to obtain the fitting equation. BioRender software was used for drawing the graphical figures.

### 2.4 Synthesis of coating materials

#### 2.4.1 Synthesis of DVB polymer and preparation of DVB/PDMS/phytase coating material

**Fig. 1:**
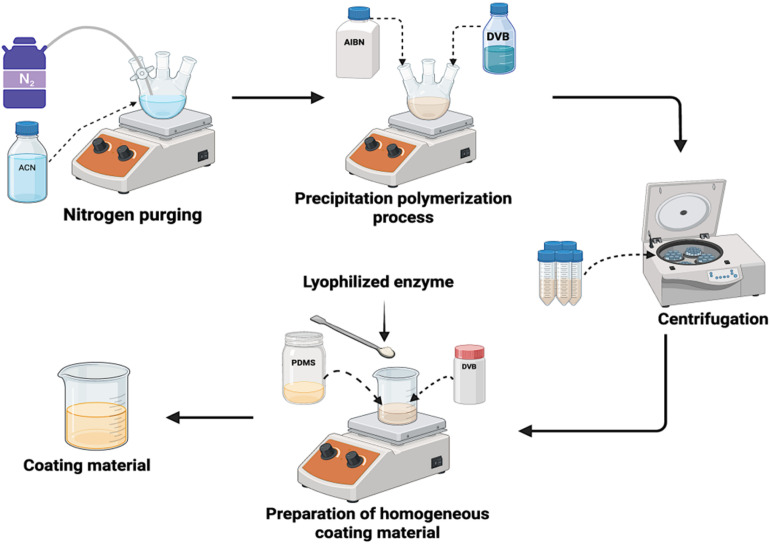
Synthesis of the DVB particles by precipitation polymerization, followed by the formulation of a homogeneous coating mixture comprising DVB, PDMS, and the phytase enzyme.

The precipitation polymerization technique was used to synthesize the DVB polymer.^40^ In a three-necked round-bottom flask, 400 mL of purified acetonitrile (ACN) was purged with nitrogen gas for 2 hours. 10 mL of the divinylbenzene (DVB) monomer was purged into the flask along with 600 mg of the 2,2-azobis (isobutyronitrile) (AIBN) initiator. It was stirred at 100 rpm and heated to 75 °C for 24 hours at room temperature. The mixture was thereafter polymerized and then centrifuged at 10 000 rpm for 30 minutes at room temperature. After discarding the supernatant, the resulting polymer particles were washed with ethanol to give DVB particles in the size of 1–5 µm by vacuum-drying. Fig. 1 is a schematic overview of the synthesis procedure. For the preparation of the DVB/PDMS/phytase coating mixture, 1 g of the synthesized DVB particles, 4 mL of PDMS, and 9 mL of hexane were added to a beaker. Additionally, 2 g of commercial phytase enzyme was incorporated into the mixture. The beaker was covered with aluminium foil and placed on a magnetic stirrer for 24 hours to obtain a homogeneous coating solution containing the enzyme. This enzyme-embedded coating material was used for further applications.

### 2.5 Fabrication of paper-based TF-SPME

**Fig. 2:**
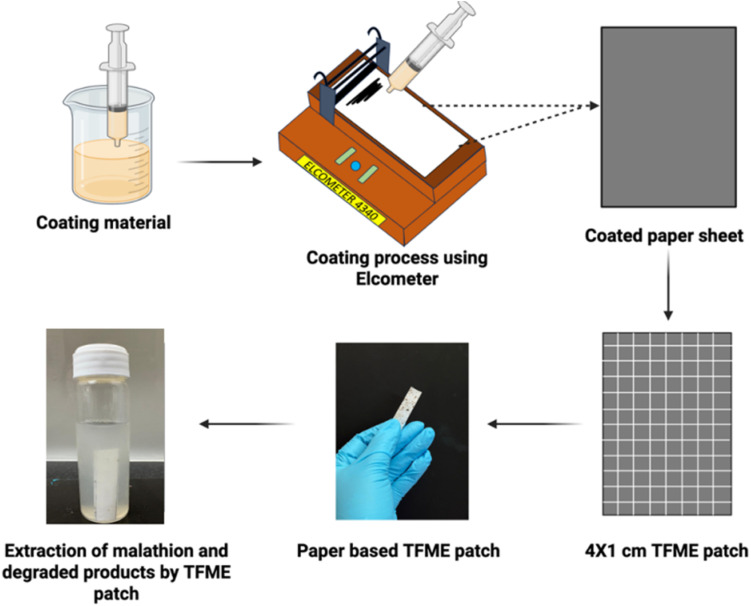
Fabrication of the paper-based microextraction patch and the extraction of malathion from the soybean seed matrix.

In preparation for the paper-based TF SPME, an A4-size paper was cleaned and placed on an automatic film applicator (Elcometer 4340), as shown in Fig. 2, with the aim of coating. For the uniform coating, the coating composition was filled into a syringe and finally applied to the paper support. The coating thickness was set to 70 µm, and the coating speed was adjusted to obtain coating uniformity on the paper substrate. The entire A4 sheet was dried in an oven after coating, and then, it was dried in the air. For the efficient extraction of analytes, the two sides of the paper were coated. The coated A4 sheet was finally trimmed into multiple 4 × 1 cm patches, which were to be used as individual sampling tools.

### 2.6 Characterization of the paper-based TF-SPME patches

Field emission scanning electron microscopy (FE-SEM) was performed using a CARL ZEISS (USA) SIGMA model with a GEMINI column, which has a resolution of 1.5 nm. Images were captured in secondary electron mode with an in-lens detector. To obtain high-resolution images, samples were examined under an accelerating voltage of 10 kV, a working distance of 5.5 mm, a magnification of 100 00×, and a resolution of one micron. Additional images were taken at 5 kV, with a working distance of 10.5 mm, a magnification of 3000×, and a resolution of 10 microns.

The energy-dispersive X-ray spectroscopy (EDX) method was used to conduct the elemental analysis of the DVB-based sorbent on a BRUKER Nano XFlash Detector (Germany). Different modes, such as a point scan, area scan, line scan, and elemental mapping modes, were used. The imaging was carried out with a magnification of 2,500×, high voltage (HV) setting, 9.8 mm working distance and a resolution of 20 µm.

### 2.7 Enzymatic degradation of malathion

The ammonium molybdate method was used to determine the phytase enzyme activity.^41^ The volume of the reaction mixture (1 mL) comprised 10 000 ppm malathion and 1 mL of the commercial phytase (1 mg mL^−1^) in a buffer. The reaction mixture was incubated at 30 °C for 32 min. An identical amount of 10 (w/v) trichloroacetic acid was added to stop the reaction. The quantity of liberated inorganic phosphate was determined by the addition of 1 mL of colour reagent that included 1% (w/v) ammonium molybdate, 3.2% (v/v) sulfuric acid and 7.2% (w/v) ferrous sulphate. A spectrophotometer was used to check absorbance at a wavelength of 700 nm. The amount of phytase activity was the quantity of the enzyme needed to liberate 1 mmol of phosphate in a minute, as indicated under the assay conditions, and it was termed as 1 unit of phytase activity.

### 2.8 Extraction of malathion using the microextraction patch

Malathion (50 ppm) was prepared as a stock solution by mixing the compound completely using a magnetic stirrer in a clear solution without any visible particulate matter. Then, the soybean seed solution was prepared. First, 30 g of homogenized soybean was added to 240 mL of deionized water and then mixed vigorously on a shaker at a temperature of 25 °C at 180 rpm for 15 min. After that, the solution was transferred to 50 mL tubes for centrifugation at 10 000 rpm for 5 min at −5 °C, and the supernatant was taken. To analyze the analyte at different concentrations, the leading malathion stock solution was diluted serially, with a concentration range of 0.5–10 ng mL^−1^. Spiking was done by adding each diluted solution to 25 mL of the soybean seed extract. The TF-SPME patch was subsequently added to each of the spiked vials and then incubated in a shaking incubator at 250 rpm to assist in the efficient extraction of malathion and its degradation product on the paper-based patch. To maximize the extraction parameters, such as stirring speed, temperature, extraction period, desorption period, and the profile of the organic solvent, the conditions of these parameters were varied. The patches were desorbed into vials containing 1 mL of acetonitrile (ACN) after extraction and then desorbed under the influence of a vortex mixer. Malathion and its degraded products, as they occurred in the soybean matrix, were quantified by GC-MS/MS analysis.

## 3 Results and discussion

**Fig. 3:**
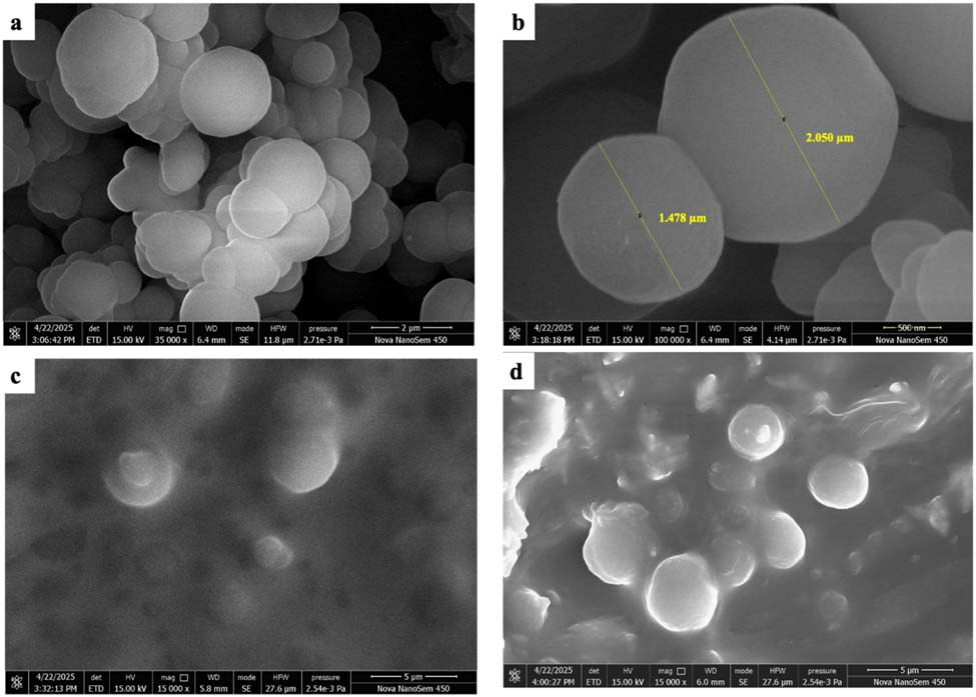
(a) and (b) FE-SEM images of the DVB sorbent particles; (c) the surface morphology of the coated paper-based patch before the malathion extraction; and (d) the coated patch after malathion extraction.

A simple paper-based microextraction tool was successfully developed for the detection of malathion from the soybean seed matrix and the degradation of the pesticide into a less toxic byproduct. To validate the synthesized sorbent materials and fabrication process, several morphological and physicochemical analyses, including FT-IR, XRD, EDX, and FE-SEM, were conducted. The FE-SEM data indicated that the size of the laboratory-prepared DVB polymers was within 1.4 µm and 2.05 µm (Fig. 3a and b). Also, it was evident from the figures that the particles were special and well distributed. This uniformity was required to get reproducible results during the experiments with the paper patches from the soybean matrix for pesticide detection.

**Fig. 4:**
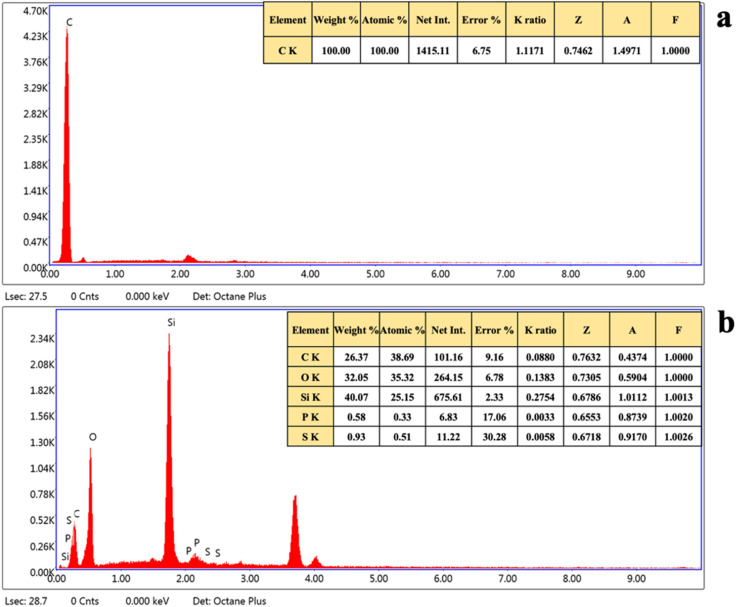
EDX of the (a) synthesized DVB particles and (b) the DVB/PDMS/phytase-coated TF-SPME patches after the extraction of malathion.

For further validation, EDX was performed for the elemental composition of the synthesized DVB particles and coated patches (Fig. 4a and b). The EDX spectrum of the DVB sorbent showed a strong peak, confirming the presence of carbon, with 100% atomic weight, which is consistent with its polymeric structure. In addition, the post-extraction paper patches demonstrated the additional elements, *i.e.*, carbon (38.69%), oxygen (35.32%), trace levels of phosphorus (0.33%), sulphur (0.51%), and silicon. The EDX analysis showed a significant transformation in the surface elemental composition after the extraction of malathion using a DVB-based patch. Although the DVB sorbent particles showed 100% carbon, the presence of phosphorus and sulphur in the post-adsorbed patches established the successful capturing of malathion pesticide using the paper patches. The presence of heteroatoms in the EDX data also ensured the strong interaction between the patch and malathion.

**Fig. 5:**
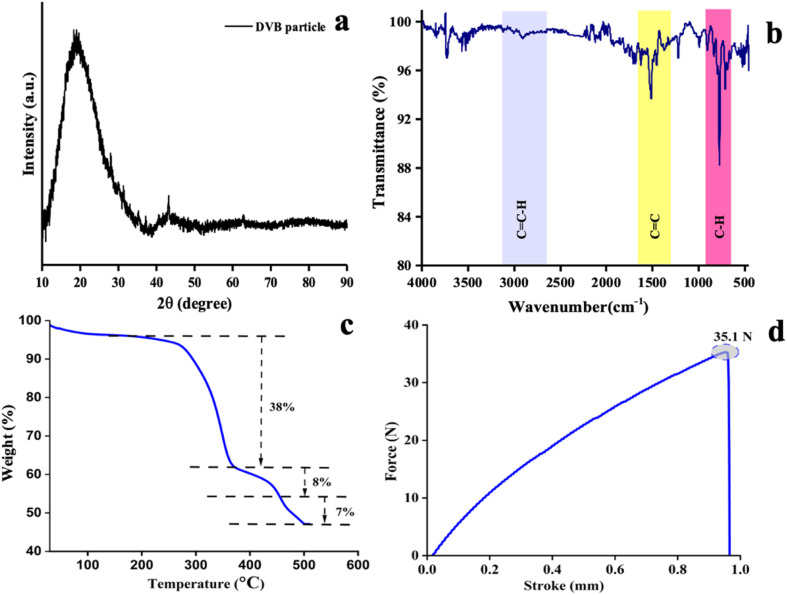
Characterization of the synthesised DVB particles using (a) X-ray Diffraction (XRD) and (b) Fourier Transform Infrared (FTIR) spectroscopy analysis. (c) Investigation of the thermal stability of the DVB/PDMS/phytase paper-based TF-SPME by TGA and (d) mechanical stability by UTM.

The structural morphology was characterized by X-ray diffraction (XRD), as shown in Fig. 5a. A broad peak of 2*θ* ≈ 220° indicated the amorphous or semi-crystalline structure. This observation also confirmed the structural morphology of the DVB sorbent particles.^42^ The FTIR spectrum (Fig. 5b) of the synthesized DVB particles revealed the characteristic C–H stretching vibration (aromatic and vinyl groups) in the range of 3100–2700 cm^−1^, aromatic C–C stretching vibrations in the region of 1700–1350 cm^−1^, and aromatic C–H bending vibrations in the range of 950–650 cm^−1^. These spectral properties validated the chemical structure of the DVB particles. The minor deviations in peak intensity and position from the literature may be due to the variability of particle sizes and the presence of trace levels of impurities that change after synthesis.

### 3.1 Stability of paper-based TF-SPME

#### 3.1.1 Thermogravimetric analysis of DVB/PDMS/phytase

Thermogravimetric analysis (TGA) was performed using Hitachi STA7200 equipment in the range of 30–500 °C to assess the thermal stability of the paper-based DVB/PDMS/phytase patches. Fig. 5c exhibits two major weight losses. The first weight loss was within the temperature range of 250–360 °C (approximately 34.22% weight loss). This significant weight loss was attributed to the thermal degradation of the patch, primarily the moisture content of the DVB patches. Another small weight loss (12.18%) occurred beyond 410 °C because of the breakdown of residual crosslinked DVB fragments and carbonaceous residues. The patch retained the residual weight (50%) at 500 °C, suggesting the formation of inorganic or carbonized residue after decomposition at high temperature. Hence, the TGA results confirmed that the DVB/PDMS/phytase-coated microextraction patch was thermally stable up to 230 °C, ensuring its robustness during the analytical sample preparation.

#### 3.1.2 Universal testing machine

To investigate the mechanical stability of the microextraction patches, the tensile property was estimated through a universal testing machine (Shimadzu-EZ-SX). It is important to know the durability and robustness of the paper patches for handling this tool for analytical applications.

The data showed that this microextraction device can withstand up to 35.26 N of tensile force, confirming the mechanical stability of the patch, and this tool can be used for this purpose without tearing and structural deformation (Fig. 5d). Therefore, this patch can maintain the functional integrity during the different stages of the sample preparation process.

## 4 Degradation of malathion from soybean seed

**Fig. 6:**
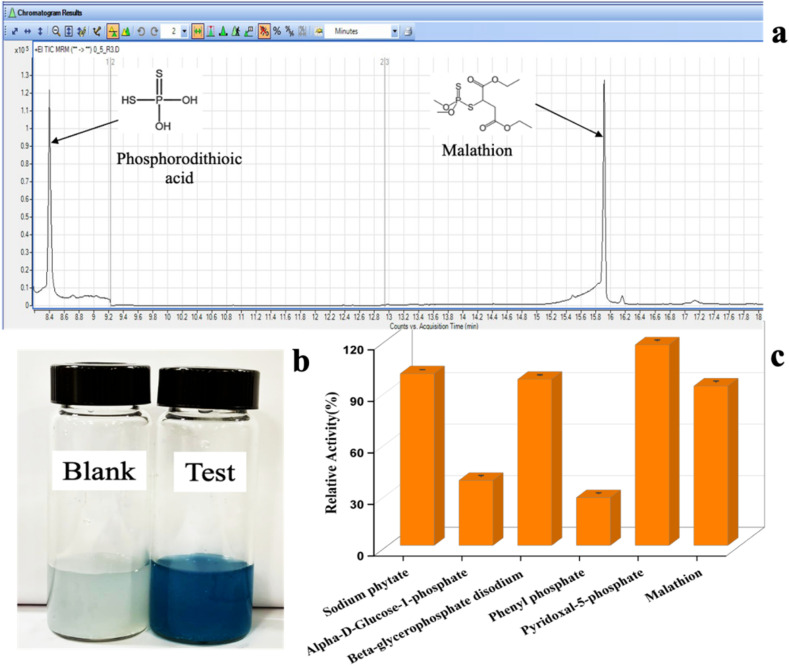
(a) The chromatogram of malathion and the degraded product, phosphorodithioic acid, from the GC-MS/MS analysis and (b) qualitative confirmation of malathion degradation. The blank has a substrate without an enzyme and the test has both an enzyme and a substrate. (c) The broad substrate specificity (promiscuity) of the phytase enzyme.

It is important to degrade the malathion into a less toxic byproduct to ensure food safety before consumption. Here, the enzyme phytase present in the paper strip catalyzed the cleavage of the malathion pesticide. The catalytic reaction produced the phosphorodithioic acid (PDA) after the malathion degradation, and this byproduct is less toxic as compared to the original pesticide. Therefore, this laboratory-designed paper patch provided dual functionality through the detection of malathion and the degradation of the pesticide in a single platform. The presence of PDA in the desorbed solution was confirmed by GC-MS/MS experiments (Fig. 6a).

For further validation, calorimetric analysis was performed. The development of a molybdenum blue complex using ammonium molybdate and inorganic phosphate in the presence of trichloroacetic acid in the reaction mixture confirmed that the enzyme can degrade malathion and release the inorganic phosphate (Fig. 6b).

The enzyme phytase was found to be promiscuous, *i.e.*, having broad substrate specificity (Fig. 6c). The maximum relative activity of pyridoxal-5-phosphate is 15% greater than that of the native substrate (sodium phytate). The hydrolysis of beta-glycerophosphate disodium also indicates the affinity of the enzyme towards structurally similar phosphate esters. Furthermore, malathion was found to progressively undergo degradation, reflecting the capability of the enzyme to act on organophosphate pesticides and showing potential activity on various substrates. Alpha-d-glucose 1-phosphate and phenyl phosphate were relatively less active. Such a wide range of substrate hydrolysis indicates the versatility of the enzyme and its possible applications both in nutritional enhancement and bioremediation.

## 5 Optimization of the analytical parameters

### 5.1 Effect of extraction time

**Fig. 7:**
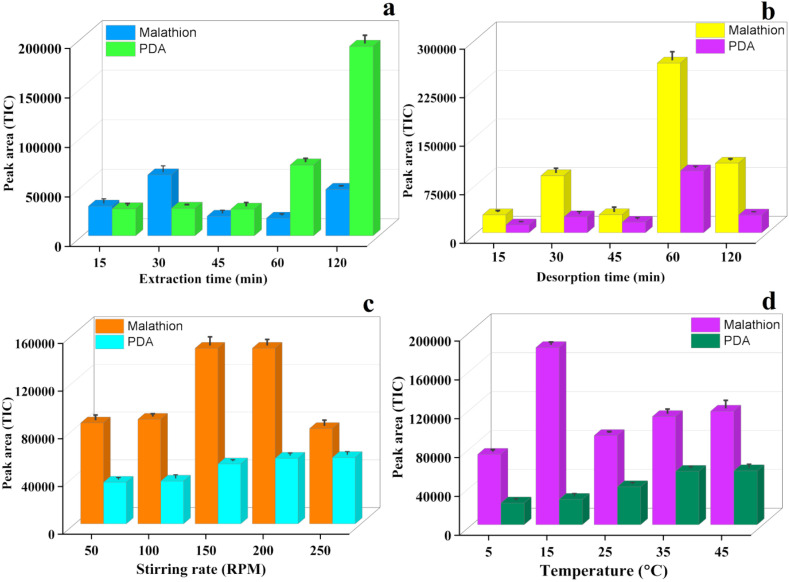
Optimization of various analytical parameters to extract malathion and PDA from the soybean seed matrix using the paper-based patches, including (a) extraction time, (b) desorption time, (c) stirring rate, and (d) temperature.

As the extraction time during sample preparation directly influenced the performance of this analytical method, the DVB-phytase-coated patch was directly exposed to the soybean seed matrices over varying durations (15–120 min). The peak area of PDA, the degraded product, showed the maximum extraction at 120 minutes (Fig. 7a). At 15 min, a moderate amount of malathion was extracted, accompanied by the early appearance of PDA, suggesting the enzymatic activity from the beginning of the extraction process. With the increase in time, the extraction efficiency of malathion did not change, indicating that the equilibrium was reached at the beginning of the extraction process. However, the amount of byproduct (PDA) increased steadily up to 120 min, suggesting the ongoing enzymatic activity of the patches on the paper.

Over time, the extracted amount of malathion did not change significantly. However, the increase in the amount of degraded product confirmed the catalytic activity of phytase on the paper-based matrix. The co-presence of DVB and phytase on a paper strip enabled dual functionality, the extraction of malathion *via* hydrophobic interactions and *in situ* enzymatic degradation. In this study, once adsorbed, the coated phytase enzyme degraded malathion, thereby producing phosphorodithioic acid (PDA). The GC-MS/MS data (peak area in Fig. 7a) showed that the PDA response steadily increased with an increase in the extraction time, supporting a post-adsorptive catalytic process.

### 5.2 Effect of desorption time

Desorption time plays an important role as it directly governs the extraction performance and recovery in the microextraction paper patches. A very short desorption time may hamper the complete mass transfer of the targeted analyte from the TF-SPME patch to the solution phase and, finally, may decrease the overall extraction efficiency. This may produce an underestimation of malathion and byproduct concentration from the soybean seed matrix. The incomplete desorption may keep the residual analyte on the patch surface, and this may influence the sensitivity of the technique.

To evaluate the optimal time, the desorption experiments were carried out with various time intervals. The results showed that a short desorption time (below 60 min) led to incomplete mass transfer from the patch to the solvent, whereas a prolonged desorption time improved the efficiency. Here, 60 min was determined as an optimal time for achieving maximum mass transfer from the substrate to the solvent matrix, and beyond this time, the performance did not significantly improve (Fig. 7b).

### 5.3 Effect of stirring rate

The stirring rate plays an important role in extraction efficiency, as the absorption and desorption rates are influenced by the degree of agitation during the sample preparation process. The increase in the stirring rate may influence the movement of malathion and its degradation byproduct from the bulk phase to the patch surface. Moreover, an increase in the stirring rate may accelerate the equilibrium process, leading to the fast extraction of the pesticide from the sample matrix.

To investigate this effect, the extractions were performed at stirring rates of 100 rpm, 150 rpm, 200 rpm, and 250 rpm. Fig. 7c shows that 200 rpm is the optimal stirring rate for the extraction and degradation of malathion from the soybean seed matrix. Beyond this point, the extraction efficiency was decreased at 250 rpm because of the short contact time between the analyte and substrate for the effective hydrolysis on the microextraction patch surface. Furthermore, a very high stirring rate during the experiments was not possible because of the physical damage to the paper patches.

### 5.4 Effect of temperature

Thermal energy influences the analyte–sorbent interaction and governs the diffusion rate during the extraction process. To evaluate the temperature dependency of the phytase/DVB/PDMS-coated patches, the microextraction tool was employed at various temperatures ranging from 5 °C to 45 °C. The data showed that the maximum amount of malathion was extracted at a temperature of 150 °C, whereas the extraction efficiency was low at 5 °C and 25 °C. A slight increase in the pesticide extraction was observed at 25 °C and 45 °C. This study showed that the optimal temperature for the extraction of malathion was 15 °C (Fig. 7d). However, the enzymatic activity of phytase to degrade the malathion was found to be optimal at 35 °C. The significant increase in the degraded products and decrease in the malathion levels at 25 °C and 35 °C confirmed that phytase actively degraded it into PDA. The increase in the degraded product and simultaneous decrease in the malathion at a specific temperature confirmed the activity of phytase for converting the malathion into less toxic PDA byproduct.^43^

## 6 *In vitro* extraction of malathion from the soybean seed matrix

The fabricated microextraction patches were directly immersed in vials containing the soybean seed samples spiked with various concentrations of malathion. The extracted patches were desorbed using acetonitrile solvent, and the analytes were analyzed by GC-MS/MS. Malathion has a log *P* of approximately 2.75. Hence, it is moderately hydrophobic and shows optimal interaction with the DVB-based matrix used on the TF-SPME patch. The hydrophobic DVB surface enhances the adsorption of malathion from aqueous samples, enabling effective extraction from the sample matrix.

**Fig. 8:**
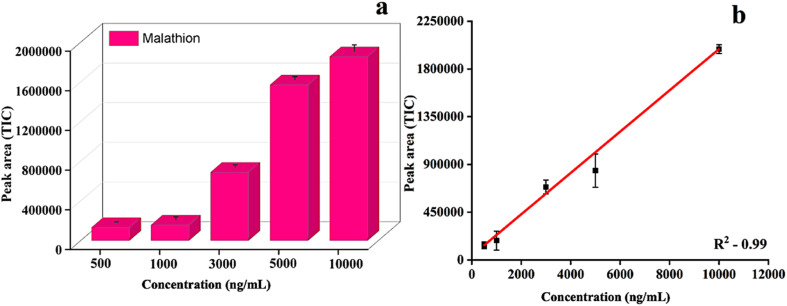
Extraction of malathion from soybean matrix (a), calibration curve of malathion extraction by DVB/PDMS/Phytase-coated paper patch (b). The fitting equation, *y* = 194.61*x* + 4160 (*R*^2^ = 0.99).

The results demonstrated the efficiency of the patches for the extraction of malathion in the environmentally relevant concentration range. The bar graph (Fig. 8a) showed that even at relatively low concentrations, the paper patches were able to capture malathion from soybean seeds. To assess the quantitative performance, a calibration curve with a series of spiked malathion in the soybean seed matrix and GC-MS/MS peak response was constructed. The fitting equation associated with the calibration was calculated as *y* = 194.61*x* + 4160 (*R*^2^ = 0.99), where *y* = peak area from GC-MS/MS analysis, and *x* = malathion concentrations (ng mL^−1^).

This equation demonstrated a strong linear correlation between the concentration and signal response, indicating the reliability of the patch for the quantitative analysis of pesticides from the soybean seed matrix. Based on this calibration model, the concentration of unknown malathion samples in soybean seed could be easily determined. This fitting equation can effectively measure the unknown concentration of malathion easily without the need for prolonged sample preparation steps. Furthermore, in SI Fig. S1, the reusability and performance of the DVB/PDMS/phytase patch for the extraction and degradation of malathion are demonstrated, and the patch can be reused up to two cycles.

The limit of detection (LOD) was calculated to be approximately ∼82 ng mL^−1^, establishing the feasibility of the technique to estimate levels of malathion as per regulatory guidelines. To further validate the performance of the patches, malathion was spiked into the soybean matrix at various concentrations. Then, the accuracy of the technique was determined to be more than 90% in most cases for estimating pesticide levels using the paper patches, confirming the robustness of the technique (Table 1). These findings emphasized the potential of the TF-SPME patch as an efficient tool for the extraction of pesticides in soybean seed samples.

**Table 1:** Accuracy test after spiking malathion standards into the soybean sample matrix

Sample	Spiked (ng mL^−1^)	Estimation by fitting the equation by our proposed method (ng mL^−1^)	Accuracy (%)
Malathion (ng mL^−1^)	500	552.4	∼89
	1000	1005.2	∼99.5
	3000	3087.5	∼97
	5000	4885.7	∼97
	10 000	10 214.4	∼97.8

The developed paper-based TF-SPME patch has shown good analytical performance and sustainability through a comparative evaluation of the system against other extraction methods that have been reported before. Traditional methods, including liquid–liquid extraction^44^ and solid-phase extraction,^45^ typically use large volumes of solvents and require relatively long times, whereas DLLME^46^ and rapid solvent extraction-gas chromatography^47^ have low LODs (0.05–0.5 µg L^−1^) but use toxic solvents and complicated methods. Contrarily, the current paper-based TF-SPME system requires 1 mL of solvent and has an SE of 4.22% and LOD of 82 ng mL^−1^, which are similar to those of more sophisticated methods like MOF-based dispersive microextraction^48^ and MIP-SPE.^49^ The technique, therefore, provides a simplified, inexpensive, and environmentally sustainable approach with high reproducibility and sufficient sensitivity to extract pesticides in solid matrices, highlighting its appropriateness in fast and field-deployable analytical applications (Table S1).

## 7 Assessment of the green analytical score of the technique

**Fig. 9:**
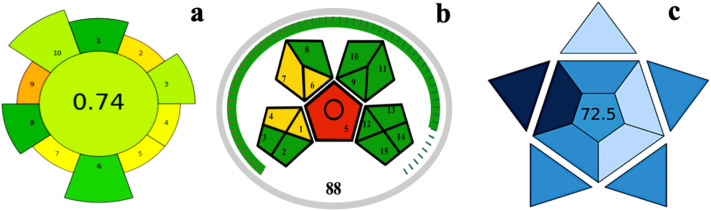
Greenness and sustainability assessment of the developed analytical method by the (a) AGREE, (b) GAPI, and (c) BAGI metrics.

To evaluate the environmental sustainability of the proposed technique, the greenness of the method was evaluated utilizing three commonly used methods (AGREE, GAPI, and BAGI). The AGREE score of 0.74 (out of 1) strongly indicates the strong compliance of the technique with Green Analytical Chemistry principles (Fig. 9a and Table S2). This method exhibited minimal solvent usage with reduced waste generation, ensuring its suitability as an eco-friendly tool for pesticide detection and degradation.

The GAPI assessment score of 88 also indicates strong compliance with the principles of Green Analytical Chemistry (Fig. 9b and Table S3). During the assessment of GAPI, most of the evaluation criteria (15 criteria) received the green index because the analysis required a low quantity of solvents and low energy consumption. A few categories showed moderate concerns (yellow), likely due to the use of small amounts of organic solvents (acetonitrile and hexane). Nonetheless, the overall profile suggests that the developed method is significantly greener compared to conventional methods for pesticide determination. Therefore, it is suitable for environmentally safe analytical workflows.

The overall sustainability of the developed paper-based enzyme-coated DVB patch method was further evaluated using the BAGI (Beyond AGREE Index) tool. The calculated BAGI score was 72.5, indicating a method with notable environmental, economic, and social sustainability. As seen in Fig. 9c and Table S4, the method performed well in areas of green chemistry compliance, analytical robustness, and social responsibility. Slightly low scores were observed in aspects related to economic factors and safety, likely influenced by the cost and handling of specific reagents. Moreover, the high BAGI score affirms that the approach is a viable, eco-friendly, and socially responsible analytical method suitable for applications in environmental monitoring.^50^

## 8 Conclusions

The present study demonstrated the successful design of a paper-based microextraction strip with enzyme phytase and divinylbenzene particles. The device was able to simultaneously detect and degrade malathion from soybean seeds. The present approach has several advantages over traditional techniques, including solid-phase extraction and liquid–liquid extraction, because it consumes a minimal amount of solvent and is cost-effective due to the use of paper for the fabrication of the sampling patches. Furthermore, the method was eco-friendly and consisted of a reduced number of sample preparation steps as compared to those in conventional methods for the quantitative estimation of pesticides. The preconcentration method was aligned with the green sample preparation method. Furthermore, this method for the quantification of malathion from soybean seeds exhibited an LOD of ∼82 ng mL^−1^, which is 24 times lower than the MRL set by regulatory Codex Alimentarius and European Union regulations. The synergistic interaction between the moderate hydrophobicity of malathion (log *P* ∼2.75) and the DVB sorbent particles present in the patch enhanced the analyte–sorbent interaction, resulting in a high extraction efficiency. Therefore, this technique may be applied for quality checks to determine food safety.

Apart from this, the presence of phytase as a coating material facilitated the degradation of malathion into less toxic phosphorodithioic acid as a byproduct, suggesting a potential disposable paper platform for detection and detoxification. Finally, it holds great potential for practical applications in environmental monitoring, food-safety analysis, and sustainable analytical technologies. Further research on the detection and degradation of malathion from naturally contaminated soybean seeds (or any real-time sample) using the fabricated TF-SPME patches is required for further validation of the developed tool. The incorporation of two properties, including the adoption of malathion by polymer sorbents and the degradation of the said pesticide by enzymatic activity, on a single analytical platform may provide a fast solution for food-safety purposes. Further research into the integration of this cost-effective disposable sampling kit with a hand-portable mass analyzer may enable the real-time estimation of organophosphate pesticides.

## Conflicts of interest

The authors declared no conflicts of interest in the study.

## Supplementary Material

RA-015-D5RA06061C-s001

## Data Availability

Supplementary information (SI) is available. See DOI: https://doi.org/10.1039/d5ra06061c.
