# Supplementary material for: Paper-based microextraction tool for the in vitro and eco-friendly detection and degradation of malathion pesticide from soybean seeds
Source: RSC Adv. 2025 Dec 2;15(55):47440–51. doi: 10.1039/d5ra06061c (PMC12670240; doi:10.1039/d5ra06061c)
Supplement: RA-015-D5RA06061C-s001 [file RA-015-D5RA06061C-s001.pdf]

Supplementary Information (SI) for RSC Advances.
This journal is © The Royal Society of Chemistry 2025

Paper-based microextraction tool for in vitro and eco-friendly detection and degradation of malathion pesticide from soybean seeds

Archana Kumari[a], Harshika Poojary[a], Chiranjit Ghosh[*], S. Balaji[*]

[a]Department of Biotechnology, Manipal Institute of Technology (MIT), Manipal Academy of Higher Education, Manipal (MAHE), Karnataka, 576104, India.

[*]Joint Corresponding authors:

Dr. Chiranjit Ghosh: chiranjit.ghosh@manipal.edu; Dr. Balaji S: s.balaji@manipal.edu

This supplementary material provides a comparative analysis that emphasizes the advantages of the proposed paper-based TF-SPME method over previously reported extraction techniques (Table S1), the reusability efficiency of the developed DVB/PDMS/Phytase patch (Figure S1), and a comprehensive evaluation of the green analytical performance of the developed paper based TF-SPME method for malathion analysis using AGREE (Table S2) , complex GAPI (Table S3) and BAGI metrics (Table S4).

1. As summarized in Table S1, the reported techniques showed wide variations in LOD (0.001 µg/L–4.0 µg/mL) and RSD (3–10%) depending on matrix type and extraction procedure. In the present study, the developed paper-based TF-SPME achieved a LOD of 82 ng/mL and an SE of 4.22%, which is similar to or less than many other traditional techniques that have been reported. Moreover, our method involves the use of minimal amount of solvent and offeres a simple, disposable, and environmentally friendly analysis. The above comparative assessment shows that the proposed method has a superior balance of analytical performance, precision, and environmental sustainability.

Table S1. Comparison of the developed paper-based TF-SPME technique with various extraction and sample preparation methods

The comparison was used to identify differences in solvent volume, extraction time, recovery rate, precision (RSD), and sensitivity (LOD) to compare the analytical performance and greenness of developed method.

	Matrix	Method	Solvent volume	Extraction time	Recovery rate	RSD	LOD	Reference
1	Drinking water	Rapid solvent extraction-gas chromatography	Methylene chloride-petroleum ether-acetone (volume ratio of 3:1:1)	6 min	98.3% to 100.9%	0.36% to 4.23%	0.05 µg/L	1
2	Agricultural water	Fluorescent Magnetic MIP	Negligible solvent (magnetic-assisted)	10 min	NA	6.5	1.26 mg/L	2
3	Human urine	DLLME coupled with HPLC–UV	Very low (microliter scale, typically 100–500 µL extraction solvent)	5 min	96.3%–101.7%	3%	0.5 µg/L	3
4	Environmental water	SPME coupled with GC–FPD	Solvent-free (fiber-based method)	30 min	75.3%–102.6%	<8%	0.049–0.301 µg/L	4

5	Fruits and vegetables	LLE coupled with HPLC–UV/VIS	High (mL scale, methanol or ACN:water 75:25 v/v)	Moderate (30–60)	NA	NA	NA	5
6	Environmental water (tap, surface, well, wastewater)	Magnetic Solid-Phase Extraction (Cu-MOF@Fe_3O_4@SiO_2) + HPLC–UV	NA	< 30 min	84.0 %–105.5 %	3.8 %–9.6 %	NA	6
7	Environmental Water	Pipette-tip micro-SPE + GC-MS	NA	NA	89.37 %–101.22 %	<6.37	0.30 ng/ml	7
8	Cherry tomatoes, broccoli, mulberries, cranberries, figs	GC-FPD	NA	NA	76.2 %–103.9%	2.1%–7.3 %	0.01–0.02 µg/L	8
9	Seven representative fruits & vegetables (potato,	Modified QuEChERS followed by GC analysis	NA	NA	84.2 %–114.4 %	NA	NA	9

	carrot, cabbage, lettuce, orange, tomato, grape)							
10	Vegetables, fruit juices, and cow's milk	Dispersive micro solid-phase extraction (D-µSPE) using ZIF-67/chitosan/Fe_3O_4/SiO_2 composite sorbent + GC analysis	10 µL Triton X-100 (dispersion agent)	NA	NA	< 4.595	0.11 ng/mL	10
11	Fruits and vegetables	Solid-phase extraction (SPE) followed by GC using acetone:ethyl acetate:hexane (10:80:10, v/v/v) as extraction solvent	NA	NA	85 %–99 %	< 5.0 %	0.005–0.01 mg/kg	11
12	Environmental	Magnetic Solid-Phase	NA	6.85 min	NA	NA	0.014	12

	samples (water/soil)	Extraction (MSPE) using Fe_3O_4/GO adsorbent followed by colorimetric detection with Cu@Ag nanoparticles					mg/L	
13	Tap water, soil, cabbage	Molecularly Imprinted Polymer Solid Phase Extraction (MIP-SPE) with GC-FPD and GC-MS/MS detection	Acetonitrile–chloroform (1:1, v/v) used as porogen (volume not specified)	NA	Tap water: 96.06–111.49%; Soil: 98.13–103.83%; Cabbage: 84.94–93.69%	3.5–9.2%	Tap water: 0.001 mg/L; Soil: 0.004 mg/kg; Cabbage: 0.004 mg/kg	13
14	Food & Water	Dispersive SPME using Cu-benzyl tricarboxylic acid MOF	2 mL	NA	≥ 92%	<10	4.0 ug/ml	14
15	Soyabean seed	Paper based SPME	1 ml	60 mins	_	_	82 ng/mL	Present work

2. Reusability test of the developed DVB/PDMS/Phytase

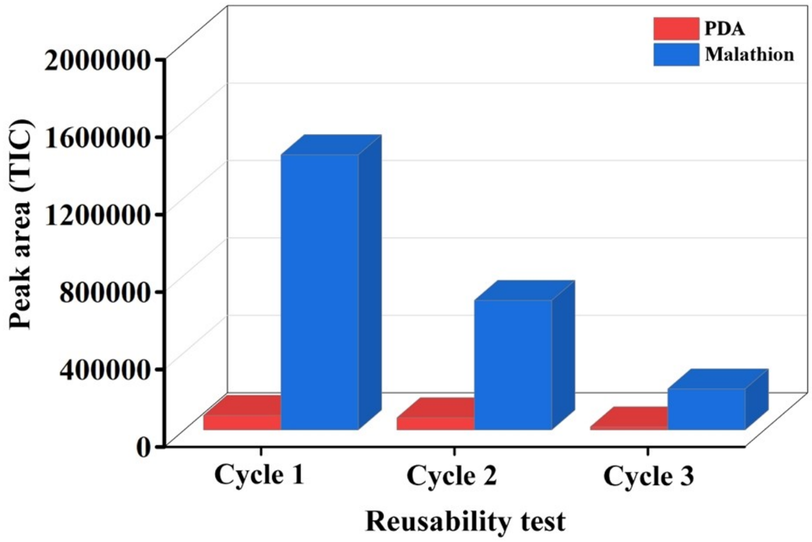

Figure S1. Reusability performance of the DVB/PDMS/phytase patch for extraction and degradation of malathion.

The bar graph represents the total ion chromatogram (TIC) peak areas of malathion and its degradation product, phosphorodithioic acid (PDA), over three consecutive extraction–degradation cycles. The gradual decrease in malathion peak area with consistent PDA detection confirms the progressive enzymatic degradation activity of the patch while demonstrating partial retention of functionality after multiple uses.

Prior to reuse, the patches were carefully cleaned and dried under a nitrogen environment to remove the impurities. The performance of the extraction was determined by the peak area of the analytes. In the case of malathion, the second cycle showed a 53% decrease in extraction efficiency than on the further decline with 86% in the third cycle. Similarly, with the degradation product of the phosphorodithioic acid (PDA), the second and third cycles had a reduction of 17% and 79%

respectively. These findings show that the patch can be reused upto two cycles. Furthermore, despite being a disposable patch, the study showed that the patch can be reused.

3.Assessment of the green analytical score of the technique

The Table S2 depict the AGREE score assessment based on 10 key principles of Green Analytical Chemistry, highlighting the method's strengths, including minimal sample preparation, in situ applicability, and waste minimization. The overall AGREE score was determined to be 0.74, with significant contributions from direct analytical techniques, avoidance of derivatisation, and multianalyte analysis. Table S3 details the green analytical assessment using GAPI metrics, where the method achieved a score of 88, demonstrating its efficiency in multi-analyte detection, sample throughput, and automation. Additionally, Table S4 summarizes the BAGI score evaluation, emphasizing the method's quantitative capabilities, miniaturized sample preparation, and environmentally conscious approach with the score of 72.5. These assessments collectively affirm the method's suitability for green analytical applications in pesticide analysis.

The environmental sustainability of the developed paper-based TF-SPME patch coated with DVB/PDMS/Phytase for the extraction of malathion from soyabean matrix was thoroughly evaluated using the GAPI software. The developed sample preparation process does not require standard storage and transport conditions. It utilizes a microextraction scale that consumes only small amounts of solvents specifically, it takes 1 mL of acetonitrile for desorption, and derivatization and mineralization treatments are not used. The method realizes a high level of efficiency, with a yield of >89% and a high purity level of >98%, following multiple green chemistry principles. The E-factor of the method is remarkably low due to minimal use of solvent and reagent, making it even greener with the total score of 83 out of 100. Table S3 summarizes Green Analytical Assessment Using GAPI Metrics. The study demonstrated excellent environmental performance, the incorporation of greener solvent alternatives to acetonitrile remains a potential step.

The Analytical Greenness Index (BAGI) score for the study has been evaluated to assess the overall environmental and operational efficiency of the method. Based on this, the study achieved

a total BAGI score of 72.5 points. The method involved quantitative and confirmatory analysis using GC-MS. The extraction process involves solid phase microextraction (SPME) technique, which enhances the efficiency and minimizes sample preparation, while utilizing commercially available reagents like acetonitrile (ACN). No additional preconcentration steps required, the method is considered highly efficient and suitable for analyzing trace level of pollutants. The semi-automated nature of the process further supports its practicality in routine laboratory analyses. The high BAGI score in reflects the balance of sensitivity, efficiency, and environmental friendliness of the developed method, making it a suitable for both environmental and analytical applications

Table S2. AGREE Score Evaluation for Green Analytical Chemistry

AGREE Principle	Description	Score
1. Sample preparation and placement	Minimizes sample preparation with a portable TF-SPME tool.	0.9
2. Hazardous materials	Uses 1 mL of ACN, a moderately toxic solvent.	0.7
3. Sustainability, renewability, and reusability of materials	> 75% of reagents and materials are sustainable or renewable	0.8
4. Waste	1Generates minimal waste (≈1 mL solvent per extraction) and the TF-SPME device can be reused	0.8
5. Size economy of the sample	Uses 25 mL of water, reducing waste.	0.7
6. Sample throughput	> 30 sample can be prepared in one hours	0.9
7. Integration and automation	The method includes only two main steps (extraction and analysis) and is semi-automated.	0.8
8. Energy consumption	GC-MS is energy-intensive (>1 KWh/sample).	0.5
9. Post-sample preparation configuration for analysis	Gas chromatography with quadrupole detection	0.9
10. Operator's safety	The use of a small quantity of ACN introduces minimal toxicity and flammability risk.	0.7

Table S3. Green Analytical Assessment Using GAPI MetricsGRE

Category	Parameter	Value

SAMPLE PREPARATION	Collection	On-line or at-line
Preservation	None	
Transport	None	
Storage	Under normal conditions	
Type of method	Extraction required	
Scale of extraction	Micro-extraction	
Solvents/reagents used	Green solvents/reagents used	
Additional treatment	None	
REAGENTS AND SOLVENTS	Amount	< 10 mL (< 10 g)
Health hazard	Slightly toxic; could cause temporary incapacitation; NFPA = 0 or 1	
Safety hazard	Highest NFPA flammability or instability score of 0 or 1, or a special hazard is used	
INSTRUMENTATION	Energy	≤1.5 kWh per sample
Occupational hazard	Hermetic sealing of analytical process	
Waste	< 1 mL (< 1 g)	
Waste treatment	Recycling	
QUANTIFICATION	Quantification	Yes

Table S4. BAGI Score Evaluation for the developed sample preparation Method

BAGI Attribute	Criteria Met	Score
Type of Analysis	Quantitative and confirmatory (GC-MS)	10
Number of Analytes Simultaneously Determined	Multi-element analysis (2–5 compounds)	5
Analytical Technique-Instrumentation	Sophisticated instrumentation (GC-MS)	5

Number of Samples that can be Simultaneously Treated	2–12 samples	5
Sample Preparation	Miniaturized extraction (SPME)	5
Number of Samples Analyzed per Hour	2–4 samples per hour	5
Type of Reagents and Materials	Common commercially available reagents (ACN)	10
Preconcentration	No additional preconcentration required	10
Automation Degree	Semi-automated with common devices	7.5
Amount of Sample	≤100 µL for bioanalytical samples (low volume water matrices)	10
Total BAGI Score	Summed Score	72.5

Reference

1 C. J. Chen JingMin, 2011, 28, 814–815.

2 Z. Ali, N. Raza, M. Hayat, L. Khezami, M. Khairy, E. A. Almuqri, N. S. Basher, H. Parveen and A. A. Chaudhary, Frontiers in Nanotechnology, DOI:10.3389/fnano.2024.1516133.

3 M. Ramin, M. Khadem, F. Omidi, M. Pourhosein, F. Golbabaei and S. J. Shahtaheri, Iran J Public Health, 2019, 48, 1893–1902.

4 Z. Yao, Talanta, 2001, 55, 807–814.

5 Zahra Batool, International Research Journal of Modernization in Engineering Technology and Science, DOI:10.56726/IRJMETS30268.

6 M. Yeganeh, M. Farzadkia, A. Jonidi Jafari, H. R. Sobhi, A. Esrafili and M. Gholami, Microchemical Journal, 2022, 183, 108082.

7 A. Esrafili, M. Ghambarian, M. Tajik, M. Baharfar and M. Tabibpour, ChemistrySelect, 2020, 5, 2966–2971.

8 Y. Liu, S. Liu, Y. Zhang, D. Qin, Z. Zheng, G. Zhu, Y. Lv, Z. Liu, Z. Dong, X. Liao and X. Li, Food Control, 2020, 107, 106754.

9 J. Dong, G. Chen, L. Zhao, Y. Feng and Y. Bian, Int J Environ Anal Chem, 2025, 105, 1297–1309.

10 M. Ghorbani, P. Mohammadi, M. Keshavarzi, M. H. Saghi, M. Mohammadi, A. Shams and M. Aghamohammadhasan, Anal Chim Acta, 2021, 1178, 338802.

11 A. Lal, G. Tan and M. Chai, Analytical Sciences, 2008, 24, 231–236.

12 F. Faghiri, M. Hajjami and F. Ghorbani, Sens Actuators B Chem, 2021, 343, 130157.

13 H. G. Zuo, J. X. Zhu, C. R. Zhan, L. Shi, M. Xing, P. Guo, Y. Ding and H. Yang, Environ Monit Assess, 2015, 187, 394.

14 M. Habila, B. Alhenaki, A. El-Marghany, M. Sheikh, A. Ghfar, Z. ALOthman and M. Soylak, J Sep Sci, 2020, 43, 3103–3109.
